# Incidental retroaortic innominate vein in a patient with acute aortic dissection

**DOI:** 10.1186/s13019-020-01318-5

**Published:** 2020-09-29

**Authors:** Hideki Sasaki, Takashi Harada, Hiroshi Ishitoya, Osamu Sasaki

**Affiliations:** 1 Department of Cardiovascular Surgery, Ehime Prefectural Central Hospital, 83 Kasuga-cho, Matsuyama, Ehime 790-0024 Japan; 2 Division of Internal Medicine, Tokyo-Shinagawa Hospital, 6-3-22, Higashi-Oi, Shinagawa, Tokyo, 140-8522 Japan

**Keywords:** Retroaortic innominate vein, Acute aortic dissection

## Abstract

**Background:**

Retroaortic innominate vein is a rare anomaly. It has been reported in patients with congenital anomalies such as Tetralogy of Fallot or right aortic arch. However, isolated retroaortic innominate vein is quite rare.

**Case presentation:**

A 63-year-old man was transferred to our institution because of Stanford type A acute aortic dissection. Incidentally, we noticed that the left innominate vein coursed under the aortic arch and was directed into the superior vena cava on computed tomography. We performed emergent hemiarch replacement.

**Conclusions:**

Attention must be paid to the cannulation site for venous uptake and the method of myocardial protection.

## Background

Retroaortic innominate vein is a rare anomaly. It has been reported in patients with congenital anomalies such as Tetralogy of Fallot or right aortic arch. However, an isolated retroaortic innominate vein is quite rare. We present a patient with a diagnosis of Stanford type A acute aortic dissection in whom a retroaortic innominate vein was incidentally found on computed tomography.

## Case presentation

**Fig. 1:**
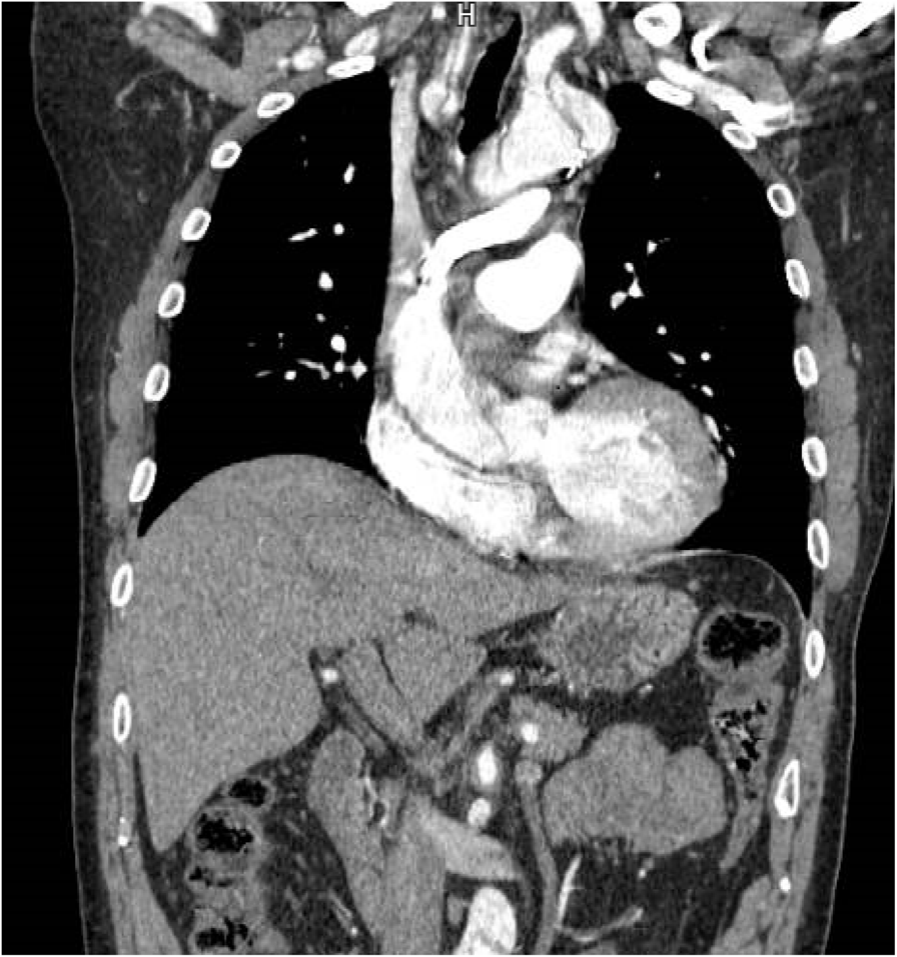
Left innominate vein courses underneath aortic arch and is directed into superior vena cava

A 63-year-old man was transferred to our institution because of Stanford type A acute aortic dissection (AAAD). Incidentally, we noticed that the left innominate vein coursed under the aortic arch and was directed into the superior vena cava (SVC) on contrast-enhanced computed tomography (CT) (Fig. 1). We decided to perform emergent surgery. Median sternotomy approach was used. After the pericardium was opened, we confirmed that the left innominate vein coursed beneath the aortic arch. We established cardiopulmonary bypass (CPB) using right subclavian and left femoral artery inflow with right atrial (RA) drainage. Both antegrade cardioplegia and retrograde cardioplegia were used for myocardial protection. A retrograde cardioplegic cannula was inserted into the coronary sinus under transesophageal echo (TEE) guidance. Hemiarch replacement was performed with hypothermic circulatory arrest (bladder temperature 25 °C). An intimal tear was found in the small curvature of the proximal aortic arch, which was excluded. Antegrade selective cerebral perfusion was commenced for brain protection. Circulatory arrest time was 51 min. Aortic cross clamp time was 117 min, with CPB time of 211 min. The subsequent postoperative course was uneventful. The patient was discharged from our institution on the 18th postoperative day. We have followed him in the outpatient clinic.

## Discussion

**Fig. 2:**
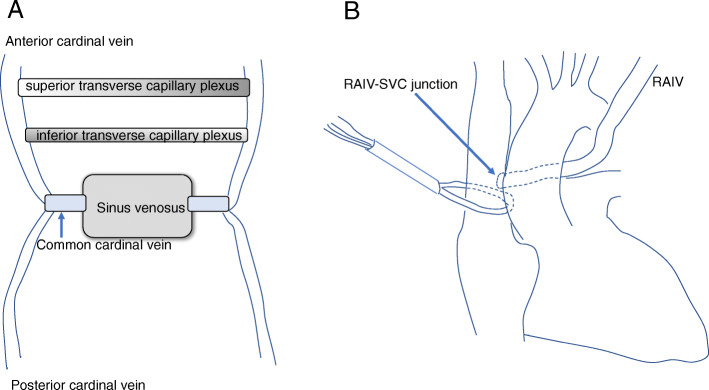
**a**. Schema of embryology. **b** If insertion of the cannula into the coronary sinus under direct vision with bicaval drainage is chosen, the SVC must be snared more caudally

The left innominate vein is usually located anterosuperior to the aortic arch, and connects with the right innominate vein, forming the SVC. However, it rarely courses via an alternative pathway. Kerschner reported the first case of an alternative course of the left innominate vein more than one hundred years ago [1]. This rare anomaly has been reported in patients with cardiac anomalies. Its incidence is 0.55% among patients with congenital heart anomaly. It is commonly related to tetralogy of Fallot or right aortic arch [2]. Cases of an isolated retroaortic innominate vein (RAIV) are extremely rare. Nagashima et al. reported that its incidence was 0.02% (1 of 4805) in patients without congenital heart anomaly [3]. During normal fetal development, systemic veins develop from paired anterior and posterior cardinal veins. They unite on each side, and form common cardinal veins. Venous return eventually directs into the sinus venosus. The anterior cardinal veins continue to the bifurcation of the internal jugular veins and subclavian veins on each side. However, a large part of the left anterior cardinal vein vanishes. Venous flow from the left side of the head and arm directs into two anastomotic plexuses (superior and inferior channels), and eventually reaches the right anterior cardinal vein by the eighth week [2]. Usually, the inferior transverse capillary plexus regresses, and the superior transverse capillary plexus becomes the left innominate vein (Fig. 2a). The left common cardinal vein becomes the coronary sinus. The oblique vein of Marshall is formed by the left anterior cardinal vein [4]. It is not clear how the RAIV is formed. A possible explanation is failure of usual development of the superior transverse anastomosis and, as a result, an alternative pathway in the caudal position remains and forms the RAIV [4].

RAIV can be problematic if contrast CT is not performed in clinical settings. It may be difficult to insert a central venous catheter from the left jugular/subclavian vein. Permanent pacemaker lead insertion from the left subclavian vein can be hazardous [5]. When surgery is performed in patients with AAAD using retrograde cardioplegia, insertion of the cannula into the coronary sinus under direct vision can be a problem. If insertion of the cannula without opening the RA under TEE guidance is used, RAIV does not cause a problem. If insertion of the cannula into the coronary sinus under direct vision with bicaval drainage is chosen, the site of SVC cannulation should be placed more caudally. Moreover, the SVC must be snared more caudally (Fig. 2b). Otherwise, blood from the RAIV returns into the RA, which makes it difficult to insert a retrograde cardioplegic cannula into the coronary sinus. Furthermore, attention should paid to avoiding injury of the RAIV when we encircling the SVC for snaring.

Although we did not perform total arch replacement (TAR) in this patient, it is required when an intimal tear is located in the aortic arch. RAIV may prevent surgeons from looking at the distal aortic arch and performing maneuvers for anastomosis. The higher possibility of injury to the RAIV should be considered while dissecting the posterior part of the aorta and aortic arch. When surgeons retract the RAIV to acquire a good operative field, laceration and subsequent bleeding may occur. When bleeding occurs from the junction between the RAIV and SVC, it might be difficult to control it. Furthermore, strong retraction for a long time can cause thrombosis in the RAIV, which might lead to catastrophic pulmonary embolism. If aortic surgery is required in patents with RAIV via left thoracotomy, the same attention is mandatory for proximal anastomosis.

## Conclusion

Attention must be paid to the cannulation site for venous uptake and the method of myocardial protection when surgery is performed in patients with acute aortic dissection and RAIV.


**Additional file 1.**

## Data Availability

Not applicable.

## Abbreviations

AAAD: Stanford type A acute aortic dissection
SVC: Superior vena cava
CT: Computed tomography
CPB: Cardiopulmonary bypass
RA: Right atrium
TEE: Transesophageal echo
RAIV: Retroaortic innominate vein
TAR: Total arch replacement
